# Genetic impact on cognition and brain function in newly diagnosed Parkinson’s disease: ICICLE-PD study

**DOI:** 10.1093/brain/awu201

**Published:** 2014-07-30

**Authors:** Cristina Nombela, James B. Rowe, Sophie E. Winder-Rhodes, Adam Hampshire, Adrian M. Owen, David P. Breen, Gordon W. Duncan, Tien K. Khoo, Alison J. Yarnall, Michael J. Firbank, Patrick F. Chinnery, Trevor W. Robbins, John T. O’Brien, David J. Brooks, David J. Burn, the ICICLE-PD study group, Roger A. Barker

**Affiliations:** 1 John van Geest Centre for Brain Repair, University of Cambridge, Cambridge, UK; 2 Department of Clinical Neurosciences, University of Cambridge, Cambridge, UK; 3 Medical Research Council, Cognition and Brain Sciences Unit, Cambridge, UK; 4 Behavioural and Clinical Neuroscience Institute, University of Cambridge, UK; 5 Computational, Cognitive and Clinical Neuroscience Laboratory, Imperial College London, London, UK; 6 Brain and Mind Institute, University of Western Ontario, London, Canada; 7 Department of Psychology, University of Western Ontario, London, Canada; 8 Institute for Ageing and Health, Newcastle University, Newcastle, UK; 9 Griffith Health Institute and School of Medicine, Griffith University, Gold Coast, Australia; 10 Institute of Genetic Medicine, Newcastle University, Newcastle, UK; 11 Department of Psychiatry, University of Cambridge, Cambridge, UK; 12 Imperial College London, London, UK; 13 Department of Clinical Medicine, Positron Emission Tomography Centre, Aarhus University, Denmark

**Keywords:** Parkinson’s disease, cognition, functional MRI, genetics

## Abstract

Parkinson’s disease is associated with multiple cognitive impairments and increased risk of dementia, but the extent of these deficits varies widely among patients. The ICICLE-PD study was established to define the characteristics and prevalence of cognitive change soon after diagnosis, in a representative cohort of patients, using a multimodal approach. Specifically, we tested the ‘Dual Syndrome’ hypothesis for cognitive impairment in Parkinson’s disease, which distinguishes an executive syndrome (affecting the frontostriatal regions due to dopaminergic deficits) from a posterior cortical syndrome (affecting visuospatial, mnemonic and semantic functions related to Lewy body pathology and secondary cholinergic loss). An incident Parkinson’s disease cohort (*n* = 168, median 8 months from diagnosis to participation) and matched control group (*n* = 85) were recruited to a neuroimaging study at two sites in the UK. All participants underwent clinical, neuropsychological and functional magnetic resonance imaging assessments. The three neuroimaging tasks (Tower of London, Spatial Rotations and Memory Encoding Tasks) were designed to probe executive, visuospatial and memory encoding domains, respectively. Patients were also genotyped for three polymorphisms associated with cognitive change in Parkinson’s disease and related disorders: (i) rs4680 for COMT Val158Met polymorphism; (ii) rs9468 for MAPT H1 versus H2 haplotype; and (iii) rs429358 for APOE-ε2, 3, 4. We identified performance deficits in all three cognitive domains, which were associated with regionally specific changes in cortical activation. Task-specific regional activations in Parkinson’s disease were linked with genetic variation: the rs4680 polymorphism modulated the effect of levodopa therapy on planning-related activations in the frontoparietal network; the MAPT haplotype modulated parietal activations associated with spatial rotations; and APOE allelic variation influenced the magnitude of activation associated with memory encoding. This study demonstrates that neurocognitive deficits are common even in recently diagnosed patients with Parkinson’s disease, and that the associated regional brain activations are influenced by genotype. These data further support the dual syndrome hypothesis of cognitive change in Parkinson’s disease. Longitudinal data will confirm the extent to which these early neurocognitive changes, and their genetic factors, influence the long-term risk of dementia in Parkinson’s disease. The combination of genetics and functional neuroimaging provides a potentially useful method for stratification and identification of candidate markers, in future clinical trials against cognitive decline in Parkinson’s disease.

## Abstract (precis)

See Dujardin (doi:10.1093/brain/awu218) for a scientific commentary on this article. Nombela *et al.* present data from the ICICLE-PD study of cognition in newly diagnosed Parkinson’s disease. Consistent with the ‘Dual Syndrome’ hypothesis, impairments in executive function reflect a frontal dopaminergic syndrome modulated by COMT genotype, while visuospatial and memory deficits reflect disruption of temporo-parietal systems modulated by MAPT and APOE.

## Introduction

Parkinson’s disease was often considered to be primarily a motor disorder although dementia has long been recognized as a feature of the condition (Gowers, 1893). More recently the early onset and heterogeneity of cognitive impairments in Parkinson’s disease have been recognized, even in the absence of dementia (Muslimovic *et al.*, 2005). The cognitive deficits of Parkinson’s disease affect visuospatial, attentional, executive and memory functions (Janvin *et al.*, 2006; Hely *et al.*, 2008; Elgh *et al.*, 2009; Aarsland and Kurz, 2010; Pedersen *et al.*, 2013) due to the combination of abnormal neurotransmitter systems (e.g. dopaminergic and cholinergic) and both cortical and subcortical Lewy body pathology (Kehagia *et al.*, 2010). We have proposed two facets of cognitive deficits in Parkinson’s disease, in a ‘Dual Syndrome’ hypothesis: (i) changes in dopaminergic transmission through the corticostriatal networks leading to deficits in planning, working memory, response inhibition and attentional control; and (ii) posterior cortical Lewy body pathology and secondary cholinergic loss affecting visuospatial, mnemonic and semantic functions (Kehagia *et al.*, 2013).

Cognitive impairments are present at diagnosis in a significant proportion of affected individuals with between 24% and 62% of newly diagnosed patients with Parkinson’s disease having deficits in executive (e.g. Tower of London Task), visuospatial (e.g. Spatial Rotations Task) or memory (e.g. Memory Encoding Task) performance compared to healthy controls (Foltynie *et al.*, 2004*a*; Williams-Gray *et al.*, 2007*a*; Elgh *et al.*, 2009; Yarnall *et al.*, 2014). By 3 years after diagnosis up to 57% of patients have frontostriatal or visuospatial deficits and 10% have Parkinson’s disease dementia (Williams-Gray *et al.*, 2007*a*) rising to 17% by 5 years (Williams-Gray *et al.*, 2007*a*), 26% by 8 years (Aarsland *et al.*, 2003), 46% by 10 years (Williams-Gray *et al.*, 2013) and 83% by 20 years (Hely *et al.*, 2008). Thus only ∼15% of patients with Parkinson’s disease remain cognitively intact in the long term (Aarsland *et al.*, 2011). It is therefore important to ascertain what determines cognitive decline, and how it relates to subsequent dementia.

Genetic factors are implicated in Parkinson’s disease cognitive impairments (Goldberg and Weinberger 2004; Morley *et al.*, 2012). For example, catechol-O-methyl transferase (COMT) is involved in cortical dopamine degradation. A common polymorphism at codon 158 (Val158Met) affects its enzymatic activity 4-fold (Chen *et al.*, 2004), and influences executive task performance in healthy individuals (Stokes *et al.*, 2011; Fallon *et al.*, 2013) and patients with Parkinson’s disease (Foltynie *et al.*, 2004*b*; Williams-Gray *et al.*, 2007*b*). The way in which the polymorphism affects cortical dopamine levels suggests that either too little or too much dopamine worsens task performance, in accordance with an inverted U-shaped curve (Goldberg and Weinberger, 2004; Williams-Gray *et al.*, 2007*b*, 2009*b*; Rowe *et al.*, 2008). Our hypothesis was that the COMT polymorphism would affect dopamine-dependent working memory and planning systems in frontostriatal networks, and introduce a non-linear (U-shaped) relationship between neurocognitive function and levodopa dose.

A second gene linked to cognitive performance and dementia in Parkinson’s disease is the microtubule-associated protein tau (*MAPT*). The *MAPT* haplotype H1 (versus H2) not only predisposes to Parkinson’s disease but also Parkinson’s disease dementia (Goris *et al.*, 2007), possibly by altering the cortical expression of 4- versus 3-repeat isoforms of tau (Williams-Gray *et al.*, 2009*a*). Our hypothesis was that fronto-parietal systems for visuospatial function, related to dementia with Parkinson’s disease, would be relatively preserved in H2 carriers versus H1 carriers.

Finally, apolipoprotein E (*APOE*) has been proposed to alter the risk of Parkinson’s disease dementia (Chen *et al.*, 2004; Huang *et al.*, 2006; Goris *et al.*, 2007; Williams-Gray *et al.*, 2009*b*; Chung *et al.*, 2012; Gomperts *et al.*, 2012, 2013) as well as being a risk factor for Alzheimer’s disease, even if it does not significantly alter the risk of developing Parkinson’s disease without Parkinson’s disease dementia (Peplonska *et al.*, 2013; Multhammer *et al.*, 2014). *APOE* has three allelic variants (*APOE2*, *3* and *4*), and *APOE4* carries the highest risk for Alzheimer’s dementia (Corder *et al.*, 1993) with *APOE2* carrying the lowest. Our hypothesis was that memory systems centred on the temporal lobe and hippocampus in particular would be most impaired in APOE4 carriers.

In this study we examined the impact of these genetic factors on cognitive function in a large cohort of patients with newly diagnosed Parkinson’s disease. We used functional MRI to measure regional brain functions during a range of tasks that encompass the main cognitive deficits reported in Parkinson’s disease (Williams-Gray *et al.*, 2007*a*; Barone *et al.*, 2011; Ekman *et al.*, 2012; Hampshire *et al.*, 2012; Winder-Rhodes *et al.*, 2013; Nagano-Saito *et al.*, 2014; Yarnall *et al.*, 2014). The results of the comprehensive neuropsychological assessment undertaken by the participants are published elsewhere (Yarnall *et al.* 2014). This neuroimaging study focuses on a set of three tasks that provide robust experimental models of important cognitive functions affected by Parkinson’s disease, including planning and working memory (Tower of London Task), visuospatial function (Spatial Rotations Task) and memory (abstract image encoding and recognition). We sought to define how the early cognitive deficits in newly diagnosed patients with Parkinson’s disease map onto changes in brain activation, and how these activations in patients varied as a function of the common genetic variations in *COMT*, *MAPT* and *APOE*.

## Materials and methods

### Subjects

The Incidence of Cognitive Impairment in Cohorts with Longitudinal Evaluation – Parkinson’s Disease (ICICLE-PD) study recruited a cohort of 219 patients with incident Parkinson’s disease from community and outpatient clinics at the John van Geest Centre for Brain Repair, Cambridge, UK (*n* = 49) and Parkinson’s Disease clinics in Newcastle-upon-Tyne/Gateshead, UK (*n* = 119) [from the ICICLE-PD cohort, 169 patients agreed to participate in the functional MRI study (separate day within 4 months from initial assessment)]. We used the United Kingdom Parkinson’s Disease Society (UKPDS) Brain Bank diagnostic criteria (Hughes *et al.*, 2002), with reconfirmation after 18 months, to diagnose Parkinson’s disease. Full inclusion and exclusion criteria are described in Yarnall *et al.* (2014). In brief, exclusion criteria were: parkinsonism diagnosed before the onset of the incidence study; insufficient working knowledge of English to perform the neuropsychological assessment; dementia at presentation [defined as Mini-Mental State Examination (MMSE) score < 24 or Diagnostic and Statistical Manual of Mental Disorders, Fourth Edition (DSM IV) criteria for dementia or Movement Disorder Society criteria for dementia]; lack of mental capacity to give informed consent under UK legislation; history of parkinsonism following the onset of cognitive impairment; history or examination suggestive of dementia with Lewy bodies, multiple system atrophy, progressive supranuclear palsy, repeated strokes or stepwise progression of symptoms indicative of ‘vascular parkinsonism’; and, exposure to dopamine receptor blocking agents at the onset of symptoms.

Unrelated age- and sex-matched controls were recruited from the MRC Cognition and Brain Sciences Unit volunteer panel in Cambridge, UK (*n* = 50) and from community sources at the Newcastle site (*n* = 35). The Local Research Ethics Committee approved the study, performed according to the Declaration of Helsinki, with all participants providing written consent.

Participants undertook a battery of standardized clinical and neuropsychological assessments including: the Unified Parkinson’s Disease Rating Scale (MDS-UPDRS) (Goetz *et al.*, 2008); MMSE (Folstein *et al.*, 1975); Montreal Cognitive Assessment (MOCA) (Nasreddine *et al.*, 2005); National Adult Reading Test (NART) (Nelson and O'Connell, 1978) estimate of premorbid IQ; verbal fluency for words starting with the letter P/F (60 s) (Benton, 1968) and semantic fluency for animals (90 s) (Goodglass *et al.*, 1972). Levodopa equivalent daily dose (LEDD) value was calculated according to Tomlinson *et al.* (2010). Patients were assessed ON their usual dopaminergic medication (Williams-Gray *et al.*, 2007*b*). Additional neuropsychological tests and the Geriatric Depression Scale-15 for depression are reported by Yarnall *et al.* (2014).

DNA was extracted from peripheral blood using standard phenol/chloroform techniques. Genotyping for rs4680 (*COMT* Val158Met), rs9468 (*MAPT* H1 versus H2 haplotype) and rs429358 plus rs7412 (*APOE* genotype 1–4) was performed using an allelic discrimination assay and run on an HT7000 detection system (Applied Biosystems).

### Experimental design

On the scanning day participants were trained for 30 min to perform the tasks and practice keyboard responses. Participants lay supine in the MRI scanner, with auditory protection and head fixation using foam-rubber pads. Stimuli were back-projected onto a screen, and viewed via a mirror on the headcoil. Three functional MRI experiments were performed.

### Tower of London Task

We used a ‘one-touch’ modified version of the Tower of London Task (Shallice, 1982; Williams-Gray *et al.*, 2007*b*), as a model of prefrontal executive function in Parkinson’s disease (Rowe *et al.*, 2001; Lewis *et al.*, 2003). The task presented with two racks of three coloured balls in different pockets. Participants determined the minimum number of moves to rearrange the balls to match the racks (Owen *et al.*, 1995; Baker *et al.*, 1996). The control task required one to count the difference in the number of balls between the two displays. Responses were made with a right hand button-box. The paradigm lasted for 10 min 46 s, with intermixed presentations of experimental and control items, cued on the screen before each trial as ‘plan’ or ‘substract’, respectively, with three levels of difficulty (levels 2, 3 and 4 according to the number of moves or number of differences in the ball count-dependent variable 2) and intertrial intervals of 5–15 s. No feedback was provided. The dependent variables were the latency of response (including mainly the thinking time plus a small contribution from the motor response time for the one-touch version of this task) and accuracy.

### Spatial Rotations Task

Spatial impairments in Parkinson’s disease are independent of executive skills (Cronin-Golomb and Braun, 1997) and depend on the integrity of posterior parietal cortex and a fronto-parietal network (Cohen *et al.*, 1996; Zacks, 2008). We used this task to probe posterior cortical function, analogous to earlier studies of Alzheimer’s disease (Jacobs *et al.*, 2012). Each item consisted of a reference pattern (5 × 5 grid, top) and four response patterns (bottom). One response pattern corresponded to the reference, after rotation by ±90° or 180°. Three randomized levels of difficulty (levels dependent variable 2, 3, 4) were defined by the complexity of the pattern. The control condition required matching the reference and unrotated response patterns. The task lasted 10 min 46 s, with alternate experimental and control items (cued on screen by ‘rotate’ or ‘match’, respectively) and intervening rest intervals of 5–15 s (cued by ‘rotate’ or ‘match’ for experimental and control items, respectively). No feedback was provided. The dependent variables were the latency of response and number of accurate responses.

### Memory Encoding Task

Memory deficits in Parkinson’s disease are most related to encoding rather than impairments in retention or retrieval processes (Bronnick *et al.*, 2011). Encoding deficits generally have a different aetiology to executive impairments (Kehagia *et al.*, 2010), and are linked to hippocampal function (Aarsland *et al.*, 2011). The Memory Encoding Task was selected accordingly. Subjects viewed abstract pictures organized in seven blocks (displayed alternatively with intertrial intervals of 5–15 s) of six images for 4 s each, with a 1 s cross-hair fixation between, and were asked to memorize them. Participants saw 30 different images in the scanner; 18 of them appeared once (exposition fold = once), 12 appeared twice (exposition fold = twice). After scanning (20-min delay), participants completed a recognition test of these 30 images, intermixed with 32 lures. They reported whether they had seen each picture before by two-alternate forced-choice button responses. No feedback was provided. The dependent variables were the response latency, the number of accurate responses and the d’ score of hit rate versus false alarms.

### MRI acquisition processing and analysis

A Siemens TIM Trio 3 T scanner (Siemens Medical Systems) was used at one site and a 3 T Philips Intera Achieva scanner at the other. Participants underwent high resolution magnetization prepared rapid gradient echo scanning (MP-RAGE: repetition time = 2250 ms, echo time = 2.98 ms, flip angle = 9°, inversion time = 900 ms, 256 × 256 × 192 isotropic 1 mm voxels). During functional MRI, ‘BOLD-sensitive’ T_2_* weighted echo-planar images were acquired (repetition time = 2000 ms, echo time = 30 ms, flip angle = 78°, 32 × 3 mm sequential descending slices, in-plane resolution 3 × 3 mm, slice separation 0.75 mm) with 320 volumes for Tower of London and Spatial Rotations Tasks and 250 volumes for Memory Encoding excluding 10 initial dummy volumes.

MRI data were processed using Statistical Parametric Mapping (SPM8, www.fil.ion.ucl.ac.uk/spm). Functional MRI data were converted from DICOM to NIFTII format, spatially realigned to the first image, and corrected for acquisition delay by sinc interpolation with reference to the middle slice. The mean functional MRI volume and MP-RAGE were co-registered using mutual information, and the MP-RAGE segmented and normalized to the Montreal Neurological Institute (MNI) T_1_ template. The normalization parameters were applied to all spatiotemporally realigned functional images and upsampled to 2 × 2 × 2 mm, before smoothing with an isotropic Gaussian kernel with full-width half-maximum of 5 mm.

Individual analysis of all three tasks was modelled with the stimulus onset times and durations per item. First level general linear modelling included six regressors: stimuli were modelled as a boxcar function per condition (experimental or control condition) and level of difficulty (2, 3 and 4) for Tower of London and Spatial Rotations Task whilst Memory Encoding was modelled including all stimulus category (pictures seen once and pictures seen twice, independently of encoding success). A parametric modulator for each trial, value 1 / reaction time, was included separately for each trial type and condition. Error trials were modelled separately. Regressors were convolved with a canonical haemodynamic response function and its first temporal derivative. Six rigid-body motion correction parameters were included as nuisance covariates. Contrast images were extracted for individuals and entered into a second level region of interest analyses. For the Tower of London and Spatial Rotations tasks, subjects were excluded if they performed below threshold, as defined by two criteria: (i) long thinking time to solve an item, defined as a latency of response >17 s [response time average + 2.5 standard deviations (SD) in the sample]; (ii) <1 correct answer per type of item (control and experimental task) and level of difficulty (2, 3 and 4 in both Tower of London and Spatial Rotations Tasks).

**Figure 1:**
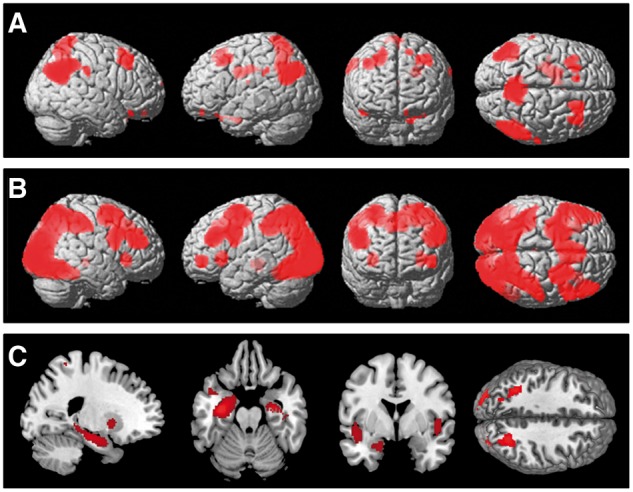
Statistical parametric maps contrasting activity in active versus baseline conditions rendered into a canonical brain in standard anatomic space. (**A**) Activity during planning minus control condition on Tower of London Task across all groups. (**B**) Activity during rotations minus baseline on Spatial Rotations Task across all groups. (**C**) Activity during encoding (pictures seen once) minus baseline on the Encoding Memory Task across all groups. Figures show areas of signal change above a threshold of *P* = 0.05 after FDR correction for the whole brain volume.

Functional MRI data were analysed by region of interest analysis at the group level (see ‘Results’ section and Fig. 1) and corrected for multiple comparisons [2-tailed significance level was set at *P* < 0.05 cluster-based false discovery rate (FDR)]. Then, region of interest analyses were performed using individual measures of averaged effect size (‘beta’ parameter estimates) for each region of interest, extracted using MarsBaR (MARSeille Boîte À Région d’Intérêt) toolbox (http://marsbar.sourceforge.net).

The independent *a priori* specification of regions of interest was based on previous studies of Tower of London and Spatial Rotations Tasks (Williams-Gray *et al.*, 2007*b*; Hampshire *et al.*, 2012). Beta values in eight *a priori* regions of interest were extracted: right dorsolateral prefrontal cortex (DLPFC), left DLPFC, right frontopolar cortex, bilateral posterior parietal cortex and precuneus. Additionally, caudate nuclei (right caudate: *x* = −10, *y* = 15, *z* = 2; left caudate: *x* = −10, *y* = 15, *z* = 2, 10 mm radius sphere) were included because of their high relevance within the frontostriatal network in mediating executive functions in healthy controls (Owen *et al.*, 1996) and Parkinson’s disease (Lewis *et al.*, 2003). A task-specific region of interest template for Memory Encoding Task (Hampshire *et al.*, 2012) was based on independent 60 healthy control data: bilateral hippocampus (right hippocampus, left hippocampus), left superior parietal gyrus, right inferior frontal gyri pars triangularis and pars opercularis, left inferior frontal gyrus, left occipital and a large region of interest including posterior temporo-parieto-occipital area.

The region of interest and behavioural analyses used SPSS (version 21). The first set of analyses used initial parsimonious ANOVAs in which categorical variables were run, including: region of interest, task condition and difficulty as within-subject factors and disease group (patients versus controls) and site (Cambridge versus Newcastle) as between-subject factors. However, several continuous cognitive and clinical variables have been shown in previous studies to affect brain function (e.g. age, disease progression, levodopa doses) (Williams-Gray *et al.*, 2007*a*; Barone *et al.*, 2011; Taylor *et al.*, 2013). We therefore ran secondary ANCOVAs to control for the possible effects of these variables. As there were many candidate variables, the optimal approach we used was a stepwise multiple linear regression approach, progressively excluding variables, variables which explained minimal variance. We started each model with entry variables of: age, sex, years of education, MMSE, MOCA, NART, letter and category fluency UPDRS-III, LEDD and duration of disease. We report both the significant contributory variables/covariates and the percentage of variance they explained.

## Results

### Demographics and neuropsychology

Gender, age, MOCA and MMSE scores were matched between groups and sites (Table 1), with no significant interactions between these factors and site. For years of education there was a main effect of site [*F*(1,172) = 23.431; *P* < 0.001] with fewer years at Site 2, and a main effect of disease group [*F*(1,172) = 19.760; *P* < 0.001] with controls having spent longer in formal education, but no significant interaction. There were corresponding differences between groups (higher score in controls) and sites (higher scores at Site 1) in terms of category fluency [disease: *F*(1,172) = 15.544; *P* < 0.001, site: *F*(1,172) = 12.392; *P* < 0.001] and letter fluency scores [disease: *F*(1,172) = 3.754; *P* < 0.054, site: *F*(1,172) = 10.735; *P* < 0.001] and an interaction between disease and site for category fluency [*F*(1,172) = 10.735; *P* < 0.001], with relatively higher scores in controls at Site 1. Patients at Site 1 had longer duration of disease [*F*(1,107) = 100.624; *P* < 0.001], and were on a higher dose of levodopa [*F*(1,107) = 48.402; *P* < 0.001] but were similar in their motor severity (UPDRS-III subscale).

**Table 1:** Demographic and clinical variables for participants in each group and site

	Control Site 1	Parkinson’s disease Site 1	Control Site 2	Parkinson’s disease Site 2	*P *Group	*P* Site	*P *Group × Site interaction
**Gender (M/F)**	27/22	25/24	17/18	57/45	0.549	0.407	0.697
**Age (years)**	63.83 ± 5.8	65.36 ± 7.9	66.23 ± 8.4	64.81 ± 11.1	0.84	0.94	0.77
**Years of education**	15.30 ± 6.1	14.02 ± 2.6	13.1 ± 3.9	13.03 ± 3.8	**0.001**	**0.001**	0.009
**MMSE**	29.48 ± 0.7	29.10 ± 0.9	29.16 ± 1.05	28.94 ± 1.1	0.193	0.193	0.074
**MOCA**	27.69 ± 1.7	26.06 ± 2.2	26 ± 5.9	26.07 ± 2.7	0.278	0.183	0.146
**NART**	121.85 ± 5.2	114.58 ± 8.6	113.5 ± 25.5	116.69 ± 9.4	0.448	0.845	0.639
**Semantic fluency**	18.53 ± 5.4	14.38 ± 4.2	12.37 ± 5.7	11.33 ± 4.6	0.054	**0.001**	0.493
**Category fluency**	31.95 ± 7.5	22.04 ± 6.2	23.12 ± 8.4	21.21 ± 8.1	**0.001**	**0.001**	**0.001**
**MDS-UPDRS-III**		29.28 ± 11.02		25.36 ± 10.7		0.06	
**LEDD**		484.56 ± 369		167.69 ± 129		**0.001**	
**Duration (months)**	Mean	21.1 ± 13.2		6.11 ± 4.7		**0.001**	
	Median	19.2 ± 13.2		4.7 ± 4.7		**0.001**	

*P-*values are presented separately for comparisons of group (Parkinson’s disease versus control), site (1 versus 2) and the interaction between site and disease, using ANOVAs (except chi-squared tests of gender). Data are shown without correction for multiple comparisons (values in bold are significant after Bonferroni correction). In view of the skewed distribution of symptom duration (Shapiro-Wilk test *P* < 0.001), the median values for duration are also show (*Mann-Whitney test *P*-value).

Table 2 compares key clinical and demographic markers for participants in the main ICICLE-PD study and those completing the functional MRI studies investigation, confirming that there were no significant differences. Table 3 shows the *COMT*, *MAPT* and *APOE* genotype distributions among patients with Parkinson’s disease.

**Table 2:** Clinical and demographic values of the ICICLE-Parkinson’s disease (Yarnall *et al.*, 2014) cohort and subgroup participating in this functional MRI study

	ICICLE-PD	Functional MRI-ICICLE
***n***	219	141
**Mean age**	65.9	65.08
**MDS UPDRS-III severity**	28.32	27.34
**MOCA**	25.70	26.06
**Male:female**	140:79	82:59

No differences were significant (χ^2^ and *t*-test contrasts between groups as appropriate).

**Table 3:** The distribution of the different polymorphisms of the studied genes (*COMT, MAPT* and *APOE*) Parkinson’s disease participants per site

Genes	Polymorphism	Site 1	Site 2	Total
***COMT***	Met/Met	15	32	47
	Met/Val	22	59	81
	Val/Val	7	30	37
***MAPT***	H1/H1	26	85	111
	H1/H2	16	34	50
	H2/H2	2	2	4
***APOE***	APOE2	28	66	94
	APOE3	11	50	61
	APOE4	5	5	10

### Tower of London Task

**Figure 2:**
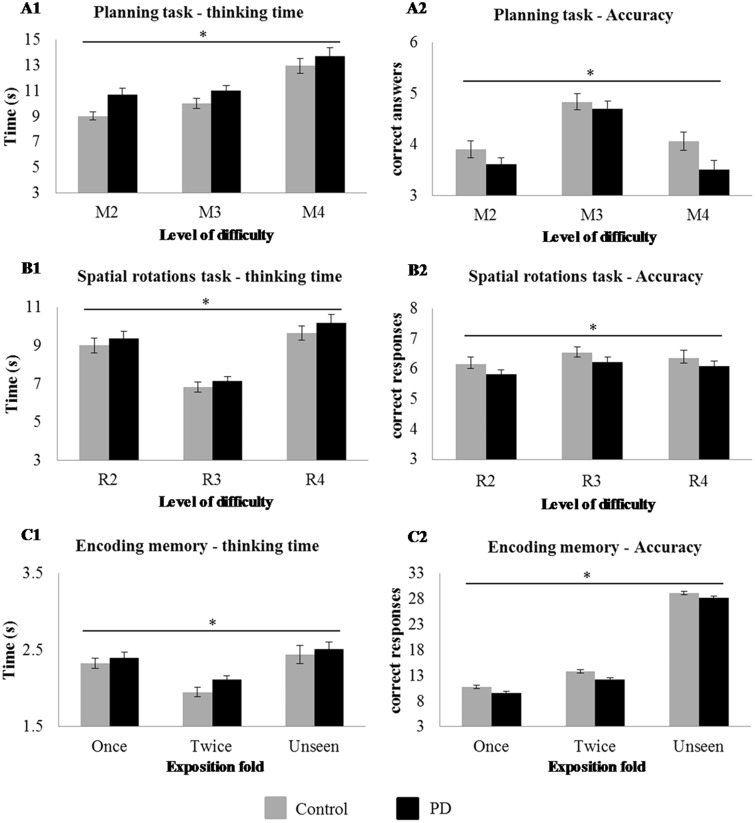
Behavioural performance by groups on (**A**) Tower of London (planning items) where difficulty is manipulated by the number of movements required; (**B**) Spatial Rotations Task (rotation items), where difficulty is manipulated by the complexity of the items to rotate; and (**C**) Encoding Memory Task, where difficulty is manipulated by the number of expositions in the memory task. **A1** and **B1** show response latency against the three level of difficulty for patients and controls. **A2** and **B2** show results in accuracy (the number of correct responses) against levels of difficulty for patients and controls. **C1** shows the number of correct, incorrect and unseen responses during the post-scan test for patients and controls. **C2** shows the number of correct responses for patients and controls, against exposure fold. (once versus twice). *Significant interaction between condition and difficulty (**A1** and **A2**), significant interaction between disease and difficulty (**B1** and **B2**), significant exposure effect (**C1**) and disease effect (**C2**), *P* < 0.05. PD = Parkinson’s disease.

Across the two sites the number of patient participants (Cambridge/Newcastle) completing the Tower of London task was *n*_Patient_ = 117 (41/76) and the number of healthy controls *n*_Control_ = 69 (43/16). Behavioural performance is illustrated in Fig. 2.

### Latency of response

The within subject factors of condition [control versus plan, *F*(1,186) = 497.432; *P* < 0.001] and difficulty [*F*(2,372) = 63.762; *P* < 0.001] were significant with an interaction effect between condition and difficulty on latency of response [*F*(2,370) = 73.744; *P* < 0.001], confirming that more difficult planning items required longer response times. Repeated-measures ANOVA confirmed an effect of site [*F*(1,186) = 7.278, *P* < 0.008, shorter at Site 1]. There was no main effect of disease (*F* < 1) or interaction effect between disease and site (*F* < 1).

The addition of between subject demographic and neuropsychological variables (age, years of education, MMSE, MOCA, NART, semantic and category fluency scores) in separate repeated-measures ANCOVAs revealed a shorter response latency in younger subjects [*F*(1,186) = 6.090; *P* < 0.015], with a higher number of years of education [*F*(1,186) = 4.033, *P* < 0.046], higher MMSE [*F*(1,186) = 19.152; *P* < 0.001], higher MOCA [*F*(1,186) = 5.378; *P* < 0.021] and greater letter fluency [*F*(1,186) = 48.06; *P* < 0.03]. No significant effects were found for NART [*F*(1,186) = 1.290; not significant] or category fluency [*F*(1,186) = 3.551; not significant].

In a separate analysis of patients with Parkinson’s disease only, the addition of disease-specific between-subject variables (UPDRS-III, LEDD and duration) in repeated measures ANCOVA indicated that patients with higher UPDRS-III score took marginally longer to respond [*F*(1,117) = 3.827, *P* < 0.05]. Neither LEDD nor duration had a significant effect. Separate repeated-measures ANOVAs with *COMT*, *MAPT* and *APOE* genotype indicated no effect on latency (*F* < 1).

A stepwise multiple regression analysis in patients with Parkinson’s disease indicated that the MMSE explained significant variance in latency in the resulting model [model *F*(4,117) = 8.395, *P* < 0.004, MMSE *t*(116) = −2.897, *P* < 0.004, 6.7% of the variance explained r = 0.26].

### Accuracy

Task condition [*F*(1,186) = 71.414; *P* < 0.001] and difficulty [*F*(2,372) = 47.9.3; *P* < 0.001] effects were significant with a significant interaction between condition and difficulty [*F*(2,370) = 16.473; *P* < 0.001] confirming that more difficult planning items were less likely to be completed. Repeated-measures ANOVA confirmed an effect of site [*F*(1,186) = 20.586, *P* < 0.001, higher in Site 1] but there was no effect of disease (*F* < 1) or interaction between disease and site on accuracy [*F*(1,186) = 2.353, not significant].

The addition of between subject demographic and neuropsychological variables in separate repeated-measures ANCOVAs showed higher accuracy scores in younger participants [*F*(1,186) = 26.075; *P* < 0.001], with more years of education [*F*(1,186) = 9.601; *P* < 0.002], higher MMSE [*F*(1,186) = 19.331; *P* < 0.001], higher MOCA [*F*(1,186) = 14.011; *P* < 0.001], higher letter fluency score [*F*(1,186) = 14.725; *P* < 0.001] and higher category fluency score [*F*(1,186) = 11.176; *P* < 0.001]. No significant effects were found for NART [*F*(1,186) = 1.216; not significant].

In a separate analysis of patients with Parkinson’s disease only, the addition of between subject clinical variables revealed no significant effect of UPDRS-III [*F*(3,117) = 3.827; not significant], LEDD, duration, *COMT*, *MAPT* or *APOE* genotype (all *F* < 1).

The stepwise multiple regression in the Parkinson’s disease group revealed a significant model [*F*(1,117) = 12.298, *P* < 0.001] of explanatory variables that included years of education [*t*(116) = 4.224, *P* < 0.001], MOCA [*t*(116) = 3.321, *P* < 0.001] and NART [*t*(116) = −2.089, *P* < 0.039] explaining a total 24.5% of the variance.

### Functional MRI regional activity

**Figure 3:**
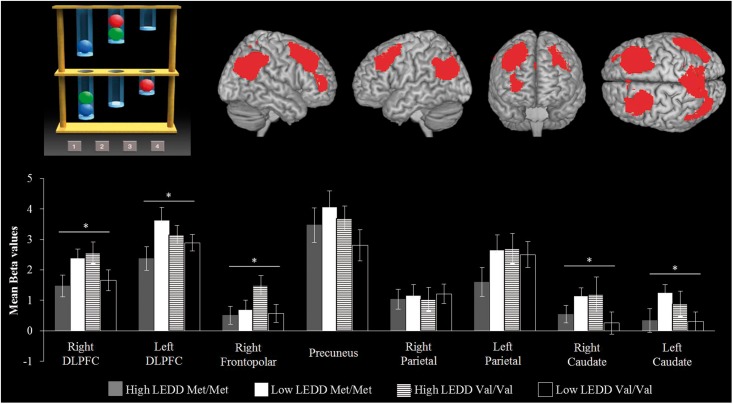
For the Tower of London Task (*top left*), the activation in regions of interest is presented separately by COMT genotype and LEDD in patients (*bottom*). The *y*-axis of each graph represents the mean activation in terms of average parameter estimates. The data are subdivided by a median split of LEDD (above versus below 275 mg/day) for each region of interest (*top right*). *P < 0.05.

The activity in regions of interest associated with planning was estimated from the contrast of ‘all planning tasks minus all control conditions’. Repeated-measures ANOVA showed no main effect of disease [*F*(1,186) = 1.353; not significant], site [*F*(1,186) = 1.723; not significant] or interaction (*F* < 1). There was a main effect of region of interest [Fig. 3; *F*(7,1309) = 130.196; *P* < 0.001] and a significant interaction between region of interest and disease group [*F*(7,1309) = 2.244; *P* < 0.029]. *Post hoc* contrast indicated that the control group had greater activation of the right frontopolar [*F*(1,186) = 6.658; *P* < 0.011], right caudate [*F*(1,186) = 11.368; *P* < 0.001] and left caudate [*F*(1,186) = 5.081; *P* < 0.025] compared to patients.

In the Parkinson’s disease group, there was a trend towards an effect of higher UPDRS-III score [*F*(1,134) = 3.359, *P* < 0.069] but no effect of LEDD (*F* < 1) or duration (*F* < 1) on activation. *COMT* genotype (contrasting Met/Met = 30 and Val/Val = 29), site and LEDD intake (median split: high LEDD >275 mg = 34, low LEDD <275 mg = 25) were used as between-subjects factors. There was no significant effect of site (*F* < 1), *COMT*, *APOE* genotypes (*F* < 1), or LEDD [*F*(1,117) = 1.387; not significant]. However, there was a significant interaction between genotype and LEDD [LEDD × *COMT*, *F*(1,117) = 5.732; *P* < 0.020] with *post hoc t*-tests confirming higher beta values in both Met/Met homozygotes at low LEDD and Val/Val homozygotes at high LEDD compared to Val/Val homozygotes at low LEDD and Met/Met homozygotes at high LEDD within the right DLPFC [*t*(58) = 2.530; *P* < 0.014], left DLPFC [*t*(58) = 2.050; *P* < 0.045], right frontopolar [*t*(58) = 2.040; *P* < 0.008], right caudate [*t*(58) = 2.089; *P* < 0.045] and left caudate [*t*(58) = 2.087; *P* < 0.040] (Fig. 3). There was no effect of *MAPT* or *APOE* genotype on activation for the Tower of London Task (*F* < 1).

### Spatial Rotations Task

Across the two sites the number of patient participants (Cambridge/Newcastle) completing the study was *n*_Patient_ = 134 (46/88) and for healthy controls *n*_Control_ = 73 (49/24). Behavioural performance is illustrated in Fig. 2.

### Latency of response

Task condition [control versus planning, *F*(1,207) = 312.534; *P* < 0.001] and difficulty [*F*(2,414) = 45.548; *P* < 0.001] along with the interaction between them [*F*(2,414) = 14.665; *P* < 0.001] were all significant, confirming that more difficult planning items required more time to be solved. Repeated-measures ANOVA revealed a significant effect of site [*F*(1,207) = 17.689; *P* < 0.001] but no effect of disease (*F* < 1) with no significant interaction (*F* < 1). There was also a significant interaction between difficulty and disease [*F*(2,414) = 2.988; *P* < 0.05], reflecting longer times to perform more difficult items by patients than controls.

There was no significant effect of age (*F* < 1), MMSE (*F* < 1), years of education [*F*(1,206) = 2.425; not significant], MOCA (*F* < 1), verbal and category fluency (*F* < 1) or NART [*F*(1,206) = 1.744; not significant] on latency of response.

For the Parkinson’s disease group, those with higher UPDRS-III scores [*F*(1,134) = 5.637, *P* < 0.019] showed longer response latencies. There was a trend towards an effect of duration [*F*(1,134) = 3.457, *P* < 0.065] but no effect of LEDD (*F* < 1), *MAPT*, *COMT* or *APOE* genotype on latency (*F* < 1).

A stepwise multiple regression in the Parkinson’s disease group revealed a minimal model [*F*(1,134) = 4.079, *P* < 0.045] including just category fluency [*t*(116) = −2.020; *P* < 0.045], which explained only 2.9% of the variance.

### Accuracy

Among within-subject factors, there was a significant effect of condition [*F*(1.207) = 179.697; *P* < 0.001] and difficulty [*F*(2,414) = 21.691; *P* < 0.001] and a significant interaction [*F*(2,414) = 66.130; *P* < 0.001]. There was an effect of site on accuracy [*F*(1,207) = 42.611, *P* < 0.001, higher in Site 1] and a trend towards a disease effect [*F*(1,207) = 3.319, *P* < 0.07, lower score in patients] but there was no significant interaction.

Accuracy was higher in younger volunteers [*F*(1,207) = 3.715; *P* < 0.05], and those with higher category fluency [*F*(1,207) = 7.264;*P* < 0.008] with weak trends for years of education [*F*(1,207) = 3.554; *P* < 0.061] and MOCA [*F*(1,207) = 3.385; *P* < 0.067], but no effects of MMSE, NART or verbal fluency [*F*(1,207) < 1.8; not significant].

**Figure 4:**
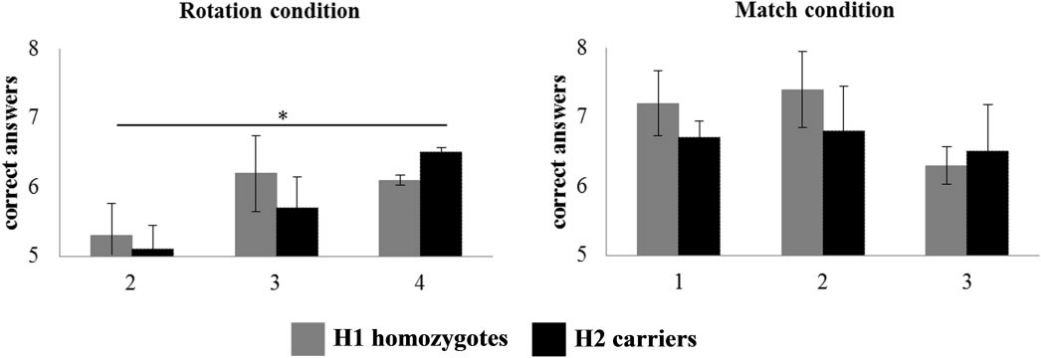
Behavioural responses in the Spatial Rotations Task, showing the number of correct responses during experimental (*left*) and control (*right*) conditions, respectively. Repeated-measures ANOVA indicated a significant interaction between MAPT (H1/H1 versus H2 carriers) and difficulty at rotation condition during the Spatial Rotations Task. *P < 0.05. Difficulty is manipulated by the complexity of the items to rotate in the Spatial Rotation Task.

The Parkinson’s disease group with lower UPDRS-III score achieved higher accuracy [*F*(1,134) = 6.839; *P* < 0.001] and there was a weak trend for shorter duration of disease [*F*(1,134) = 6.839; *P* < 0.079] but no effect of LEDD [*F*(1,134) = 2.702; not significant] or *MAPT*. A significant interaction between *MAPT* and difficulty [*F*(2,134) = 39.135; *P* < 0.001] was found, confirming that H1 haplotype homozygotes achieved lower accuracy in the more difficult items (Fig. 4). There was no significant effect of *COMT* or *APOE* genotype (*F* < 1).

The stepwise multiple regression in the Parkinson’s disease group revealed an explanatory model [*F*(2,134) = 12.317, *P* < 0.001] that included years of education [*t*(116 = 4.115; *P* < 0.001] and age [*t*(116) = −2.501; *P* < 0.014], which explained 15% of the variance.

### Functional MRI regional activity

To determine brain regions specifically activated by the rotational task, ‘all rotation events minus baseline conditions’ were analysed. Repeated-measures ANOVA showed no effect of site, disease or interaction effects between disease and site (all *F* < 1). The regions differed in their activity as revealed by a main effect of region of interest [*F*(7,1428) = 85.004; adjusted *P* < 0.001] and there was a significant interaction between site and region of interest [*F*(7,1428) = 3.374; adjusted *P* < 0.001] and between disease and region of interest [*F*(7,1428) = 1.998; *P* < 0.05] such that controls achieved greater activation than patients in a subset of regions of interest. *Post hoc t*-tests analysis showed that significant effects were localized to the left parietal [*t*(207) = 1.917; *P* < 0.05] and precuneus [*t*(207 = 2.241; *P* < 0.026].

**Figure 5:**
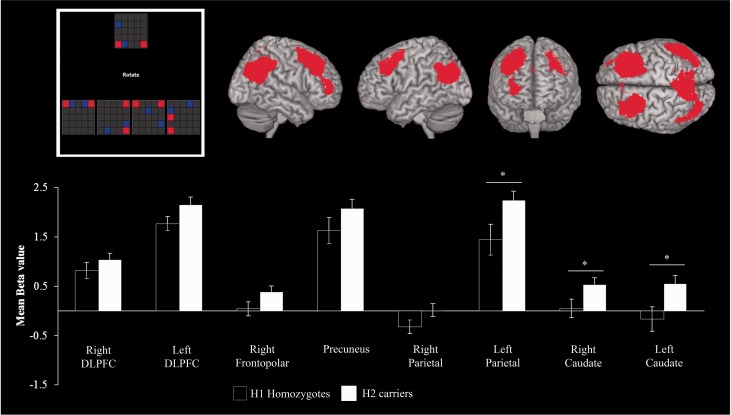
For the Spatial Rotations Task (*top left*), the activation within each region of interest (*top right*) is plotted separately for H1 patient homozygotes and H2 patient carriers. The *y*-axes represent the mean parameter estimate, in arbitrary scaled units. See text for details of the gene by region interaction. *Post hoc t*-test analysis indicated that region of interest and MAPT genotype interaction occurred at marked areas (*bottom*). *P < 0.05.

In the Parkinson’s disease patient group, the addition of between-subject variables (UPDRS-III, LEDD and duration) in separate repeated-measures ANCOVAs indicated a significant effect of LEDD [*F*(1,134) = 1.696; *P* < 0.041] but no significant effect of UPDRS-III or duration (all *F* < 1) on region of interest activity. Subsequent repeated-measures ANOVA including *MAPT* genotype and site as between-subject factors confirmed an effect of *MAPT* on beta activity within the regions of interest [*F*(1,134) = 6.600; *P* < 0.011, Fig. 5]. *Post hoc t*-test analysis indicated that H2 carriers reached significantly higher values than H1 homozygotes in the right caudate [*t*(134) = 4.045; *P* < 0.047], left caudate [*t*(134) = 6.215; *P* < 0.014] and left parietal [*t*(134) = 5.343; *P* < 0.023, Fig. 5]. There was no effect of *COMT* or *APOE* on region of interest activation during the Spatial Rotations Task (*F* < 1).

### Memory Encoding Task

Across the two sites the number of patient participants (Cambridge/Newcastle) completing the encoding memory was *n*_Patient_ = 128 (41/87) and for healthy controls *n*_Control_ = 80 (48/32). Behavioural performance is illustrated in Fig. 2.

### Latency of response

The within-subjects factor of exposure fold (once versus twice) was significant [*F*(1,208) = 62.401; *P* < 0.001]: in both groups latency of response was shorter for pictures exposed twice than for pictures exposed once. Repeated-measures ANOVA revealed significant effects of site [*F*(1,208) = 46.070; *P* < 0.001], but no disease effect or interaction between site and disease on latency.

There were no effects of age [*F*(1,208) = 1.203; not significant], MMSE [*F*(1,208) = 1.293; not significant] years of education (*F* < 1), letter fluency (*F* < 1), category fluency (*F* < 1), MOCA [*F*(1,208) = 1.501; not significant] or NART (*F* < 1). In patients with Parkinson’s disease, UPDRS-III (*F* < 1), LEDD [*F*(1,128) = 2.402; not significant], duration (*F* < 1), *COMT*, *MAPT* or *APOE* (*F* < 1) had no significant effect on latency of response. A stepwise multiple regression model [*F*(1,128) = 14.245, *P* < 0.001] indicated that duration of disease [*t*(128) = −3.774; *P* < 0.001] explained 12.4% of the variance.

### Accuracy

There was a main effect of site [*F*(1,208) = 22.476; *P* < 0.001, higher at Site 1] and disease [*F*(1,208) = 4.165; *P* < 0.043] on accuracy, indicating more recognized pictures by controls than patients, but there was no interaction between disease and site (*F* < 1). The exposure fold (once versus twice) affected accuracy [*F*(1,208) = 170.973; *P* < 0.001], in both patient and control groups with no interaction between disease and site. See Fig. 2 for details. Further analysis including d’ scores per participant indicated higher scores for controls for both pictures seen once [*t*(208) = 2.937; *P* < 0.004] and for those seen twice [*t*(208) = 3.524; *P* < 0.001].

There was no significant effect of age [*F*(1,208) = 1.203; not significant], MMSE [*F*(1,208) = 1.293; not significant], years of education (*F* < 1), MOCA [*F*(1,208) = 1.501; not significant], letter fluency (*F* < 1), category fluency (*F* < 1) or NART (*F* < 1) on encoding memory task.

In the Parkinson’s disease group, there was no significant effect of LEDD [*F*(1,128) = 2.402; not significant], UPDRS-III (*F* < 1), duration (*F* < 1), *COMT*, *MAPT* or *APOE* genotype on accuracy. The stepwise multiple regression analysis in patients revealed no single significant explanatory variables for accuracy variance.

### Functional MRI regional activity

The contrast between correctly encoded pictures ‘seen once’ minus baseline was used for repeated-measures ANOVA of regional activation. There were significant effects of site [*F*(1,208) = 226.369; *P* < 0.001] and effect of disease [*F*(1,208) = 6.050; *P* < 0.15] with higher beta values in controls and in Site 1. There was an interaction between site and disease [*F*(1,208) = 22.878; *P* < 0.01]. The regions differed in the magnitude of activation [main effect of region of interest, *F*(7,1260) = 11.920; *P* < 0.001] with an interaction between region of interest and site [*F*(7,1260) = 68.392; *P* < 0.001, higher at Site 1] and interactions between region of interest and disease [*F*(7,1260) = 9.729; *P* < 0.001]. *Post hoc t*-tests revealed significantly lower activations in patients within the left hippocampus [*t*(207) = −1.792; *P* < 0.048], left inferior frontal gyrus [*t*(208) = −4.587, *P* < 0.001], right inferior frontal gyrus pars triangularis [*t*(208) = −4.896, *P* < 0.001], right inferior frontal gyrus pars opercularis [*t*(207) = −3.333, *P* < 0.001], left parietal [*t*(180) = −4.139; *P* < 0.001], left occipital [*t*(207) = −7.056; *P* < 0.001] and temporo-parieto-occipital areas [*t*(207) = −5.008; *P* < 0.001].

There was a significant effect of MOCA on accuracy [*F*(1,207) = 4.959; *P* < 0.028] but not age (*F* < 1), years of education [*F*(1,207) = 2.262; not significant], MMSE (*F* < 1), letter fluency [*F*(1,207) = 2.187; not significant] or category fluency scores (*F* < 1) or NART (*F* < 1) .

**Figure 6:**
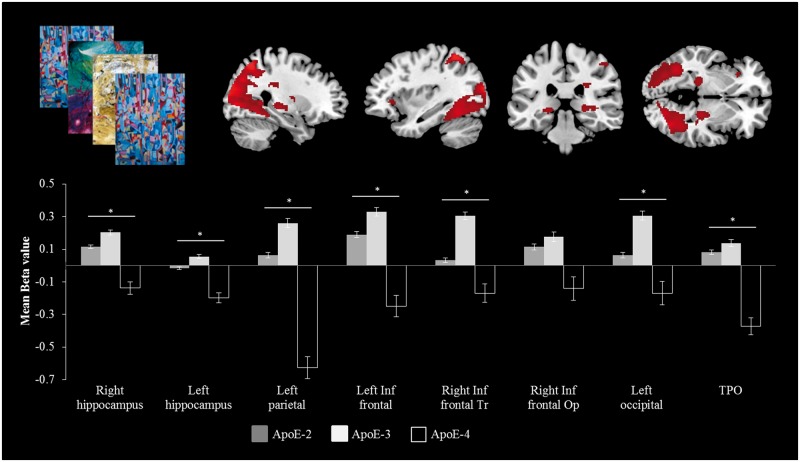
Regional activation during encoding of items in the Encoding Memory Task (*top left*), illustrating the significant interaction between regional activation and APOE genotype in Parkinson’s disease patients (see text for details). The *y*-axes represent the mean parameter estimate, in arbitrary scaled units. *Post hoc t*-test analysis indicated that region of interest and APOE genotype interaction occurred at marked areas (*bottom*). *P < 0.05. TPO = temporo-parieto-occipital.

In the Parkinson’s disease group, there was an effect of LEDD [*F*(1,107) = 7.992; *P* < 0.006] but no effect of UPDRS-III or duration (all *F* < 1) on regional activity. The addition of between subject variables (LEDD) in a repeated-measures ANCOVA revealed an interaction between region of interest and *APOE* genotype [*F*(14,609) = 1.422; *P* < 0.05], with *APOE4* carriers manifesting lower activation. *Post hoc t*-test analysis showed that the effect was focused on right hippocampus [*t*(107) = 1.866, *P* < 0.048], left hippocampus [*t*(107 = 2.635, *P* < 0.01], right inferior frontal gyri pars triangularis [*t*(107) = 2.739, *P* < 0.007], left inferior frontal gyrus [*t*(107 = 2.623, *P* < 0.01], left parietal [*t*(107 = 2.498, *P* < 0.01], left occipital [*t*(107 = 2.784; *P* < 0.007] and temporo-parieto-occipital areas [*t*(107 = 2.702, *P* < 0.008] (Fig. 6). There was no significant effect of *COMT* or *MAPT* genotype in region of interest activity during the Encoding Memory Task (all *F* < 1).

In summary, our data showed a longer latency of response (Spatial Rotations Task) and lower accuracy (Spatial Rotation and Encoding Memory Tasks) in patients with respect to controls. Score differences were stressed by demographical (age and years of education), neuropsychological (verbal fluency, MMSE and MOCA) and clinical (UPDRS-III, duration and LED) covariates. Patient impairments were reflected in brain functional measures: (i) working memory performance interacted with *COMT* polymorphisms and LEDD; (ii) spatial abilities was particularly impaired in H1 homozygotes (*MAPT*); and (iii) encoding abilities engaged lower beta values as a function of *APOE* polymorphisms.

## Discussion

The principal results of this study, in line with our hypotheses, were that (i) soon after diagnosis, neurocognitive changes are evident in fronto-striatal and parieto-temporal systems; and (ii) common polymorphisms in the *COMT*, *MAPT* and *APOE* genes are associated with differences in regional brain activity associated with executive, visuospatial and memory functions, respectively. Our results demonstrate a significant impact of these genes on cortical activity associated with cognitive tasks, either alone or through an interaction with dopaminergic medication. This study goes beyond previous work, not only in the power afforded by the cohort size, but also in its emphasis on early disease, with patients being scanned within a median of 5 and 19 months from diagnosis at the two sites, respectively—namely within 2 years of their diagnosis.

Our cohort was also representative of Parkinson’s disease soon after diagnosis: our 168 patients did not differ in their demographic variables from the larger ICICLE-PD cohort of 219 patients from which they were recruited (Yarnall *et al.*, 2014). In ICICLE-PD, the patients’ age, UPDRS-III, cognitive abilities and years of education were similar to previous large studies of community acquired cohorts in the UK undertaken in the last decade (Foltynie *et al.*, 2004*a*, *b*; Williams-Gray *et al.*, 2007*a*, 2009*a*; Elgh *et al.*, 2009; Fallon *et al.*, 2013).

### Tasks and cognition

Although Parkinson’s disease is associated with dysfunction of the fronto-striatal circuits supporting executive systems (Owen *et al.*, 1992; Kehagia *et al.*, 2010), recent evidence indicates multiple affected domains (Janvin *et al.*, 2006; Hely *et al.*, 2008; Elgh *et al.*, 2009; Aarsland and Kurz, 2010; Pedersen *et al.*, 2013). The dynamic nature of neurodegeneration, neurotransmitter loss and progressive neuropathology led to the Dual Syndrome hypothesis of cognitive deficits in Parkinson’s disease (Goris *et al.*, 2007; Kehagia *et al.*, 2013; Winder-Rhodes *et al.*, 2013): frontostriatal dopaminergic dysfunction impairs planning, working memory, response inhibition and attention control, while posterior cortical pathology and cholinergic deficits impairs visuospatial, mnemonic and semantic functions.

Our choice of functional MRI tasks succeeded in making differential demands on fronto-striatal and temporoparietal systems for planning, spatial rotation and memory (Grant *et al.*, 2013; Hampshire *et al.*, 2013). The Tower of London Task is an executive task that requires planning and working memory, which recruits a frontoparietal network that includes the prefrontal associative cortex (DLPFC) and posterior parietal cortex (Owen, 1998; Owen *et al.*, 1998; Rowe *et al.*, 2000, 2001). At all stages of Parkinson’s disease, impairments on this task have been reported with longer response times, reduced accuracy and poor neural efficiency with respect to age-matched controls (Owen *et al.*, 1992; Owen, 1998; Perfetti *et al.*, 2010) and regional impairments identified by functional MRI and PET (Baker *et al.*, 1996; Owen *et al.*, 1996; Williams-Gray *et al.*, 2007*b*). Lesion studies have confirmed that this task requires the integrity of the prefrontal cortex (Bor *et al.*, 2006) whereas pharmacological interventions and withdrawal indicate dopamine dependence (Cools *et al.*, 2002).

There was evidence of dopamine dependent Tower of London deficits in some patients, with a non-linear relationship between cortical dopamine tone and regional activation indicated by the significant LEDD by *COMT* interaction. Specifically, prefrontal cortex and caudate nuclei were more activated in Met/Met homozygotes on low-dose dopaminergic medication and Val/Val homozygotes on high-dose medication. This interaction is predicted by the inverted ‘U-shaped function’ relating dopaminergic tone and function, by which either too high or too low dopaminergic tone impairs working memory and executive performance (Goldberg and Weinberger, 2004; Williams-Gray *et al.*, 2007*b*; Rowe *et al.*, 2008; Cools and D'Esposito, 2011; Fallon *et al.*, 2013).

Our second task required mental spatial rotation, emphasizing visuospatial functions. Impairments in this domain are predictive of dementia in Parkinson’s disease (Williams-Gray *et al.*, 2009*b*). Neuroimaging of similar spatial rotations tasks in healthy adults indicates posterior parietal activation (Corballis, 1997) and prefrontal activation (Selemon and Goldman-Rakic, 1988; Goldberg and Weinberger, 2004). Parkinson’s disease increases response latencies and errors on this task (Lee *et al.*, 1998; Amick *et al.*, 2006), and reduces posterior parietal activation (Crucian *et al.*, 2003). We replicated both effects, more so in *MAPT* H1 homozygotes.

The final task involved required visual episodic memory encoding. This task evokes hippocampal and medial temporal lobe activity during encoding in healthy controls (Dove *et al.*, 2006), which we replicated. We found that even in the early stages of Parkinson’s disease, a reduction was seen in the neocortical activation associated with this task, although the magnitude and direction of hippocampal effects was similar (Fig. 6). Parkinson’s disease– mild cognitive impairment and later stages of Parkinson’s disease impair episodic memory (Weintraub *et al.*, 2004) although the relationship of early poor memory performance to the development of Parkinson’s disease dementia is unclear (Williams-Gray *et al.*, 2009*b*). Memory impairment is associated with reduced hippocampal volume in Parkinson’s disease (Davidson *et al.*, 2013; Pereira *et al.*, 2013) as well as in early Alzheimer’s disease (Sahakian *et al.*, 1988), supported by objective measures of impaired memory encoding (Weintraub *et al.*, 2011; Beyer *et al.*, 2013).

### Genetic influences on cognitive systems in Parkinson’s disease

We examined common polymorphisms that modulate the behavioural and neural consequences of Parkinson’s disease. *COMT* regulates prefrontal cortical dopamine metabolism (Chen *et al.*, 2004) and influences macroscopic cortical structure (Rowe *et al.*, 2010). Both functional MRI (Rowe *et al.*, 2008; Williams-Gray *et al.*, 2008; Fallon *et al.*, 2013) and F-DOPA PET (Wu *et al.*, 2012) studies have shown significant functional consequences of the Val157Met polymorphism in Parkinson’s disease.

The *COMT* effect is complex, with modulation by both levodopa therapy and task demands (Williams-Gray *et al.*, 2007*b*, 2009*a*). Both the *COMT* genotype and dose of extrinsic dopaminergic medication follow a non-linear U-shape function for a given task, with either too-high or too-low frontal cortical dopamine levels adversely affecting cognitive performance and activation (Rowe *et al.*, 2008). Consistent with the proposed dopaminergic modulation of frontostriatal circuits, the interaction between *COMT* genotype and LEDD was significant in dorsolateral and frontopolar prefrontal cortices and caudate nuclei.

However, some studies do not find evidence for *COMT* modulation of frontal dopamine function. For example, no interaction between *COMT* genotype and Tower of London performance was reported by Hoogland *et al.* (2010) or between *COMT* and prefrontal activation by Stokes *et al.* (2011). In Hoogland *et al.* (2010) a different Tower of London version was used (Foltynie *et al.*, 2004*b*), and no functional MRI was conducted, perhaps limiting the sensitivity to an effect of *COMT*. Interestingly, there was an interaction between LEDD and *COMT* on verbal reasoning consistent with a genotype interaction with dopaminergic medication to influence frontal cognitive ability in Parkinson’s disease. Stokes *et al.* (2011) applied a similar MRI Tower of London version to ours, but in fewer subjects and healthy middle-aged controls. Here, the ICICLE-PD data from a larger sample corroborate the *COMT* genotype modulation of frontostriatal function early in the course of Parkinson’s disease.

A second gene of interest was *MAPT*. The H1 haplotype increases the risk of developing Parkinson’s disease, and the risk of early Parkinson’s disease dementia (Goris *et al.*, 2007; Williams-Gray *et al.*, 2009*a*). Here we show that H1 carrier patients were less accurate with difficult spatial rotations, and sustained less activity in the parietal cortex and caudate nuclei (Williams-Gray *et al.*, 2009*a*), essential areas for spatial rotations (Harris *et al.*, 2000). Others have argued that there is no relationship between *MAPT* haplotype and visuospatial performance (Goldberg and Weinberger, 2004; Ezquerra *et al.*, 2008; Rowe *et al.*, 2008; Morley *et al.*, 2012), which was the case here for easy items. Our hypothesis is that as Parkinson’s disease progresses, the difference between H1 and H2 haplotype will emerge but initially only for more difficult visuospatial tasks. Our data suggest that the posterior cortical functions underlying spatial rotations task performance are not significantly regulated by dopamine, in support of the dual syndrome hypothesis.

The third gene of interest was *APOE*. During memory encoding, we found reduced brain activity within the temporo-parietal network and impaired performance in carriers of *APOE4*. Although the number of *APOE4* carriers was small, this observation is consistent with the literature (Pulkes *et al.*, 2011; Domenger *et al.*, 2012; Federoff *et al.*, 2012; Peplonska *et al.*, 2013; Multhammer *et al.*, 2014). It has been suggested that *APOE4* Parkinson’s disease carriers present more severe cortical atrophy (Wakabayashi *et al.*, 1998; Li *et al.*, 2004) and more frequent cognitive decline than patients without an *APOE4* allele (Irwin *et al.*, 2012). Our data are the first to suggest that *APOE4* also influences brain activity in the caudate nuclei, hippocampus and posterior cortical areas during a memory encoding task in recently diagnosed patients with Parkinson’s disease, a result that is in agreement with studies of Alzheimer’s disease (Bookheimer and Burggren, 2009).

The specificity of gene × task interactions suggests a contrast between *COMT*/dopamine effects on frontostriatal networks for working memory and executive function, versus *MAPT*/*APOE* modulation of temporo-parietal systems engaged in visuospatial and mnemonic functions. Other genetic factors are likely to contribute to cognitive function (Caccappolo *et al.*, 2011; Chung *et al.*, 2012), but our data clearly support a role for *COMT*, *MAPT* and *APOE* in early disease expression, and possibly disease onset (Goris *et al.*, 2007). The influence of these genetic variants is not necessarily specific to Parkinson’s disease, and we saw in the introduction how they have been associated with risk, imaging and cognitive performance differences in several neurological and psychiatric disorders. However, the variation of these three genes appears to alter the neural substrates for major cognitive domains even soon after diagnosis of Parkinson’s disease, which we suggest is directly relevant to their modification of the risk of cognitive impairment or dementia in the context of Parkinson’s disease (*APOE4*, *MAPT*) and the potentially deleterious effects of high dose levodopa therapy on some aspects of cognition in a subset of patients (*COMT*). The mechanisms of these genetic influences may include pharmacological interactions at the synapse (especially for *COMT* in relation to cortical dopamine transmission). However, they may also include neuroplasticity consequences of *COMT*, *APOE* and *MAPT* functional polymorphisms in the context of Parkinson’s disease pathogenesis, or developmental effects even if these diminish with older age (e.g. for *COMT*) (de Frias *et al.*, 2005; Starr *et al.*, 2007; Rowe *et al.*, 2010).

### Limitations

The large size of ICICLE-PD and the systematic recruitment methods have obvious advantages, but there remain methodological and inferential limitations with this study. Even with 168 participants, the non-significant results of genetic variance or LEDD may result in type II error. Our statistical methods prioritize type I errors, especially with respect to the functional MRI studies. Moreover, we suggest that more subtle effects of genotype, medication or other clinical-demographic factors may emerge with disease progression. We also rely on clinical diagnostic criteria, Although we are relatively protected against potential misdiagnosis as ICICLE-PD relies on reapplying the clinicopathologically validated diagnostic criteria after 18 months, and this is expected to be >90% accurate.

Several performance and imaging results differed between sites, despite the same research protocol (Yarnall *et al.*, 2014). Site differences are unlikely to reflect fundamental differences in the onset, risks or pathology of Parkinson’s disease. The site differences were not restricted to socioeconomic and cognitive measures, but also included the interval from diagnosis to scanning, and the levodopa dose equivalent at the time of scanning. Interestingly, the difference in UDPRS-III motor signs severity was not significant suggesting that local treatment decisions were effectively managing what may have been differential progression of the underlying disease between sites over time. Although there may be some genetic variation between northern and eastern England, we suggest that it is more likely that the differences between sites arise from different referral pathways and treatment practises. We fortunately obtained control participant data from both sites, to reduce the potential impact of regional differences in culture, genetics, education, prior health and access to care services. Socioeconomic and educational norms may influence some cognitive score differences between sites, but the sites remain comparable on the most important demographic and cognitive tests metrics (age, gender, MMSE, MOCA, NART). Most importantly for the interpretation of the regional activations, the behavioural data in the functional MRI tasks did not differ between sites. It remains to be seen whether geographical factors continue to affect the cognitive and neural markers as disease progresses, or whether the sites converge over time as their differential delay to participation gradually becomes a smaller fraction of the total disease duration.

We did not find many significant or large group effects in terms of behavioural measures. This may at first seem disappointing, given the behavioural deficits that emerge in studies of patients with more advanced disease. However, the lack of major effects in terms of behavioural data provides more relevance to the significant differences between patients and controls in the functional imaging: functional MRI may be more sensitive to the factors that modify the function of neural systems than the cognitive performance that depend on those systems at least at early stages of the disease; and the specificity of region by group interactions also raises the possibility that at early stages of the disease, compensatory mechanisms can allow for a normal performance. It also reduces the ambiguity in interpreting functional MRI data that otherwise arises if there are marked behavioural differences such that activation differences could be the cause or consequence of altered behaviour (Price and Friston, 1999; Poldrack, 2007).

This study is focused on the early presentation of Parkinson’s disease, with a median time from diagnosis to inclusion of 8 months. The genetic and clinical factors that we identify might be used to study earlier or pre-manifest states in future studies which would also avoid issues of treatment effects. However, this was beyond the scope of the ICICLE-PD study. The potential interaction between genetic variants and the rate of cognitive decline following presentation of Parkinson's disease in the ICICLEPD cohort (without dementia at presentation) will require longitudinal investigation which will be the subject of future research papers.

## Conclusion

This functional imaging study in ICICLE-PD revealed that soon after diagnosis, there are already changes in brain function and cognitive performance in patients with Parkinson’s disease. The regional activations associated with three major cognitive domains interact with genotype in the context of Parkinson’s disease. Even recently diagnosed patients had impaired performance and altered regional brain activity in three tasks that spanned frontostriatal and parieto-temporal systems. The anatomical, functional, genetic and behavioural data support the dual syndrome hypothesis for Parkinson’s disease cognition, with (i) an executive syndrome that is frontally mediated, dopamine-dependant and modulated by *COMT* genotype; versus (ii) a temporo-parietal system subject to *MAPT* and *APOE*, but not dopaminergic modulation, that is required for visuospatial and memory tasks.

## Abbreviations

DLPFC: dorsolateral prefrontal cortex
ICICLE-PD: Incidence of Cognitive Impairment in Cohorts with Longitudinal Evaluation – Parkinson’s Disease
LEDD: levodopa equivalent daily dose
MMSE: Mini-Mental State Examination
MOCA: Montreal Cognitive Assessment
NART: National Adult Reading Test
